# Niche modelling and landscape genetics of the yellow‐legged hornet (*Vespa velutina*): An integrative approach for evaluating central–marginal population dynamics in Europe

**DOI:** 10.1002/ece3.70029

**Published:** 2024-07-24

**Authors:** Cayetano Herrera, M. Alice Pinto, Mar Leza, Iris Alemany, José A. Jurado‐Rivera

**Affiliations:** ^1^ Department of Biology (Zoology) University of the Balearic Islands Palma Balearic Islands Spain; ^2^ Centro de Investigação de Montanha Instituto Politécnico de Bragança Bragança Portugal; ^3^ Laboratório Associado Para a Sustentabilidade e Tecnologia Em Regiões de Montanha (SusTEC) Instituto Politécnico de Bragança Bragança Portugal; ^4^ Department of Biology (Genetics) University of the Balearic Islands Palma Balearic Islands Spain

**Keywords:** environmental suitability, genetic structure, invasive species, microsatellite, single introduction, *Vespa velutina*

## Abstract

Genetic diversity is an important biological trait for a successful invasion. During the expansion across a new territory, an invasive species may face unprecedented ecological conditions that will determine its demography and genetic diversity. The first record of the yellow‐legged hornet (*Vespa velutina*) in Europe dates back to 2004 in France, from where it has successfully spread through a large territory in the continent, including Italy, Spain and Portugal. Integrative approaches offer a powerful strategy to detect and understand patterns of genetic variation in central and marginal populations. Here, we have analysed the relationship between genetic diversity parameters inferred from 15 *V. velutina* nuclear DNA microsatellite loci, and geographical and environmental drivers, such as the distance to the introduction focus, environmental suitability and distance to native and invasive niche centroids. Our results revealed a central–marginal dynamic, where allelic richness decreased towards the edge of the expansion range. The low environmental suitability of the territories invaded by marginal populations could prevent a diverse population from establishing and reducing the genetic diversity in populations at the expansion edge. Moreover, Markov chain Monte Carlo analysis showed both geographical and environmental distances were influencing population genetic differentiation. This study highlights the importance of combining genetic analysis with geographical and environmental drivers to understand genetic trends of invasive species to new environment.

## Graphical abstract

We tested central–marginal population dynamics on the yellow‐legged hornet in Europe. Allelic richness decreased towards the edge of the expansion range to low environmental suitability areas. Both geographical and environmental distances were influencing population genetic differentiation.

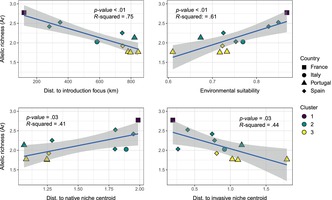

## 1 INTRODUCTION

Biological invasions are a key component of global change and represent one of the main causes of biodiversity loss (Carpenter et al., 2018), large economic costs for society (Bacher et al., 2018) and a threat to human health (Mazza et al., 2014). Consequently, alien species introductions are a problem of increasing concern (Sardain et al., 2019) and constitute a priority research area in international global change programmes (Essl et al., 2020; Latombe et al., 2022; Roura‐Pascual et al., 2021).

Understanding why some introduced alien species succeed and become invasive while others fail constitutes a major challenge in invasion ecology. Some of the most commonly studied traits underlying the success of an invasive event are related to community structure (David et al., 2017), niche conservationism (Broennimann et al., 2007), resource availability and competition (González et al., 2010), biotic resistance and community diversity (Guo et al., 2015), phylogenetic, functional or ecological originality (David et al., 2017), and founding propagule as a source of genetic diversity (Lockwood et al., 2005).

Genetic diversity plays a key role in alien species persistence and resilience in a new environment (Hoffmann & Willi, 2008). Propagule pressure during the introduction stage (including the number of introduction events and the number of introduced individuals) as well as selection pressure acting during the establishment stage can affect the genetic diversity of an invasive population (Lockwood et al., 2005). Moreover, the interplay between genetic with demographic history or ecological conditions may determine habitat suitability for species spread (Bacon et al., 2017; Unglaub et al., 2015; Williams et al., 2021) and establishment (Hewitt, 2000), and could leave a genetic footprint in populations (Stuart et al., 2021).

Ecological niche modelling represents a powerful approach to obtain quantitative spatial data regarding the habitat suitability of a given region and, hence, to assess how the potential occurrence of a given species varies across the territory (Alvarado‐Serrano & Knowles, 2014). In addition, it can be applied to detect patterns of genetic variation based on population distributions (Moussalli et al., 2009), and estimate the similarity of niches between populations with different genetic clusters (Acevedo‐Limón et al., 2020). Moreover, habitat suitability information can be useful to detect potential migration paths or isolation events (McRae, 2006), and therefore determine gene flow between populations.

The central–marginal hypothesis predicts that reduced habitat suitability and smaller effective population sizes at the marginal ranges will lead to a decline in genetic diversity from the centre to the edges of a population's range (Eckert et al., 2008; Guo, 2014; Trumbo et al., 2016). This hypothesis aligns with the concept of the abundant centre hypothesis, where habitat suitability, population abundance, and connectivity among populations decrease towards the edges of the range (Eckert et al., 2008). Consequently, genetic diversity values are expected to decrease along the expansion front due to cumulative genetic drift effects linked to successive founder events (Excoffier et al., 2009; van Boheemen et al., 2017). Biological invasions provide a unique opportunity to test these evolutionary hypotheses, as they allow the examination of genetic diversity patterns within a species' expanding range (Guo, 2014). Further research, potentially including simulation studies, is needed to disentangle the effects of environmental factors and expansion dynamics on genetic diversity.

The yellow‐legged hornet (*Vespa velutina*) is the first invasive Vespidae predator introduced from Asia to Europe (Monceau et al., 2014), where it has successfully spread from a single introduction in France in 2004 (Arca et al., 2015; Budge et al., 2017; Dillane et al., 2022; Granato et al., 2019; Herrera et al., 2023; Husemann et al., 2020; Jones et al., 2020; Quaresma et al., 2022). This invasive organism provides a unique study model for testing the central–marginal hypothesis (Guo, 2014; Trumbo et al., 2016) for several reasons: (i) its arrival in Europe is very recent (Haxaire et al., 2006), (ii) the location of the first introduction is known (Lot‐et‐Garonne, France) (Haxaire et al., 2006), (iii) there is evidence of a single founder event (Arca et al., 2015; Herrera et al., 2023; Quaresma et al., 2022), and (iv) it has successfully spread and established in neighbouring countries, such as Italy, Spain and Portugal (Laurino et al., 2020). Here, we test the central–marginal hypothesis in invasive *V. velutina* populations from France, Italy, Spain and Portugal. In addition, we identify the geographical and environmental drivers that better explain the observed genetic patterns. To that end, we assess the relationship between genetic diversity parameters, inferred from 15 nuclear DNA microsatellite loci, and geographical and environmental drivers such as the distance to the introduction focus, environmental suitability, and distance to native and invasive niche centroid.

## 2 MATERIALS AND METHODS

### 2.1 Microsatellite dataset

The microsatellite dataset was generated using adults of *V. velutina* previously genotyped in Europe with information regarding to 15 microsatellite loci for each individual (Arca et al., 2015; Herrera et al., 2023; Quaresma et al., 2022). To enable dataset merging, we checked that allele scores of each microsatellite locus were harmonised between laboratories, as described in Quaresma et al. (2022) and Herrera et al. (2023). Although the studies of Budge et al. (2017) and Jones et al. (2020) also generated *V. velutina* microsatellite data, we opted for the conservative approach of not including these data in our analyses because they were not harmonised with other studies. To have an estimate from Herrera et al. (2023) amplification error rate, 23 randomly selected samples were genotyped twice and fragment lengths were determined using GENEMAPPER 5.0 (Applied Biosystems) and checked manually. Further scoring errors due to null alleles and allelic dropout were assessed using MICRO‐CHECKER v.2.2.3 (Van Oosterhout et al., 2004).

### 2.2 Genetic structure and diversity

The Mallorca island population sample from Herrera et al. (2023) was removed from the microsatellite dataset since the geographical isolation of this territory might restrict gene flow among populations, thereby influencing the genetic parameters due to genetic drift (Wright, 1938). We initially checked population genetic analyses based on the 15 microsatellite loci obtained for the 389 individuals sampled in France (*N* = 83), Italy (*N* = 11), Spain (*N* = 104) and Portugal (*N* = 191) (Arca et al., 2015; Herrera et al., 2023; Quaresma et al., 2022). Population structure was analysed using the fast maximum‐likelihood genetic clustering approach (Beugin et al., 2018). This method is similar to the model implemented by STRUCTURE (Pritchard et al., 2000) but allows for a much faster estimation of genetic clusters by means of the Expectation–Maximisation (EM) algorithm. We initially investigated the number of cluster by using the *k*‐means algorithm, where the preferred number of clusters was evaluated using the Bayesian information criterion (BIC) score.

Based on clustering analysis, the 389 individuals were segregated into nine populations, distributed as follows: France (1), Italy (1), Spain (5) and Portugal (2). We further divided the Portuguese sample (*n* = 191) into three populations, following the north–south spread: Portugal N (north), Portugal C (centre) and Portugal S (south). Accordingly, the ensuing analyses were based on 11 different populations (Table 1) (Figure S1), whose relatedness among individuals in the same population was calculated using the estimator described by Wang (2002), including bias correction for sample size, with 1000 pairs and iterations using the R package *Demerelate* version 0.9.3 (Kraemer & Gerlach, 2017). We detected 43 pairs of individuals with high relatedness values (relatedness > 0.9), indicating that there may be nestmates, half‐siblings, or full siblings. For this reason, a single individual was kept in the analysis of those pairs of individuals with values greater than 0.9. Based on relatedness analysis, we obtained a final microsatellite dataset from 357 individuals of *V. velutina* (Table S1) and we recalculate population structure as describe above.

**TABLE 1:** Genetic parameters, geographical and environmental drivers from *Vespa velutina* populations studied. Number of individuals (N.ind), allelic richness (Ar), relatedness (Rness), the distance to the introduction focus (Dist.km), environmental suitability (Suitability), and distance to both native (Dist.nat) and invaded niche centroids (Dist.inv).

ID	Country	Cluster	N.Ind	Ar	Rness	Dist.km	Suitability	Dist.nat	Dist.inv
France1	France	1	83	2.775	‐0.050	119.215	0.869	1.987	0.194
Catalonia2	Spain	2	16	2.526	0.040	348.876	0.849	1.803	0.402
Basque_Country2	Spain	2	21	2.419	0.123	280.327	0.827	1.949	0.744
Asturias2	Spain	2	11	2.032	0.416	586.792	0.753	1.799	0.267
Galicia2	Spain	2	23	2.254	0.272	743.932	0.725	1.288	0.779
Galicia3	Spain	3	29	1.923	0.419	747.016	0.723	1.261	0.808
Italy2	Italy	2	11	2.026	0.406	583.251	0.769	1.890	0.918
Portugal2	Portugal	2	5	2.133	0.366	822.228	0.673	1.060	1.158
PortugalN	Portugal	3	57	1.773	0.543	781.259	0.733	1.078	1.027
PortugalC	Portugal	3	52	1.755	0.581	796.754	0.717	1.247	1.106
PortugalS	Portugal	3	49	1.767	0.564	845.070	0.610	1.246	1.791

Allelic richness (Ar) was estimated using the R package *pegas* (Paradis, 2010), using the rarefaction method described by (Hulbert, 1971). Finally, genetic differentiation among populations was estimated based on the pairwise F_ST_ values computed in ARLEQUIN 3.5.2.2 (Excoffier et al., 2005).

### 2.3 Collection of occurrence data

We obtained species occurrence data from the native and invaded ranges available in GBIF (accessed on 29 April 2024). Moreover, we carried out an extensive bibliographic review in search of occurrences present in papers published in English. Some of these occurrences only mentioned the location where individuals were found (village, city or region), so locations were georeferenced through Google geocoding API which returns geo‐coordinates centred of a particular parcel. Furthermore, we included the occurrences of the individuals used in previous genetic studies (Arca et al., 2015; Herrera et al., 2023; Quaresma et al., 2022) (see Table S1 from Figshare in Data Accessibility and Benefit‐Sharing section). We found 114,909 occurrences obtained from GBIF, and 2937 occurrences obtained from 15 research papers found during the literature review (Table S1).

We applied spatial thinning, a process in which a subset of locations is selected to reduce spatial autocorrelation and sampling bias, especially at larger geographical scales. Spatial thinning has been shown to decrease model overfitting and improve performance in studies where the presence records exhibit selection bias (Boria et al., 2014). In particular, we removed presence records based on a minimum neighbour distance (~9.3 km, the resolution of environmental variables explained in the next section) between the remaining records in each grid, and based on flight capacities of *V. velutina* (Sauvard et al., 2018). Then, we classified total occurrences in native or invasive distribution based on (Smith‐Pardo et al., 2020). This procedure resulted in a total of 8413 occurrences for the invasive population and 800 occurrences for the native population.

### 2.4 Geographical peripherality and environmental suitability

The first field record of *V. velutina* was reported from the French department of Lot‐et‐Garonne in 2004 (Haxaire et al., 2006). The Euclidean distance (in km) between each sampled individual and the first record in Lot‐et‐Garonne was then averaged to obtain a population‐level distance to the introduction focus.

We used ecological niche models to infer the distribution of suitable environments for *V. velutina* in Europe (Villemant et al., 2011) using global occurrence data, ensuring coverage of the full environmental niche and avoiding underestimation (Broennimann & Guisan, 2008). Climatic variables were included in our modelling analyses since they are considered the main contributors to species niche delimitation at large scales (Luoto et al., 2007). Climatic data were extracted from the WorldClim 2.1 database (Fick & Hijmans, 2017) as 5 arcmin grids (~9.3 km^2^).

Random points were sampled as background points around where *V. velutina* occurs, applying a 300‐km buffer. The background buffer was selected since the maximum spread distance registered for this species has been 78 km (Robinet et al., 2017). This procedure resulted in a total of 2600 background points for the invasive population and 4427 background points for the native population. We merged both invasive and native population datasets for further analysis.

Multicollinearity among the variables was assessed, and four uncorrelated predictors with a variance inflation factor lower than four were selected: Max Temperature of Warmest Month (bio5), Annual Precipitation (bio12), Precipitation of the Driest Month (bio14) and Precipitation Seasonality (Coefficient of Variation) (bio15). Ten per cent of the dataset was used for automated hyperparameter optimisation of the models (tuning) (Feurer & Hutter, 2019). To assure that a particular method is the best for all situations, it is recommended to include different models from different modelling techniques (Hirzel & Le Lay, 2008; Johnson & Gillingham, 2005). Hence, we selected 10 different models: Generalised Linear Models (GLM), Generalised Additive Models (GAM), Multivariate Adaptive Regression Spline (MARS), Classification Tree Analysis (CTA), Flexible Discriminant Analysis (FDA), Artificial Neural Network (ANN), Random Forest (RF) and Generalised Boosting Method (GBM), available in *biomod2* R package (Thuiller et al., 2009). We fitted and evaluated the models in Europe using weighted background, as described in Barbet‐Massin et al. (2012). Each model was fitted with 70% of the data and evaluated with the remaining 30% using ROC cross‐validation. We applied this methodology 20 times to each single model. Finally, we ensembled each single model using ROC > 0.7. The final ensemble model at the sampled‐individual level was then averaged to obtain population‐level environmental suitability.

We performed niche quantification, comparison and divergence tests using *ecospat* R package (Broennimann et al., 2020; Di Cola et al., 2017). Niche availability was defined using background environmental data, while niche occupancy was defined using occurrence data for invasive and native populations with a 100‐pixel resolution for the grid of environmental space. Moreover, we estimated Schoener's D as an index of niche overlap between populations using *ecospat* R package, which ranges between 0 (no overlap) and 1 (full overlap). Likewise, we performed niche divergence test (equivalency and similarity) using 1000 replications. The niche equivalency test determines whether niches of two entities in two geographical ranges are less equivalent than random (i.e. whether the niche overlap is not constant when randomly reallocating the occurrences among the native and invasive ranges). On the other hand, the niche similarity test addresses whether the environmental niche occupied in one range is less similar to the one occupied in the other range than would be expected by chance (Warren et al., 2008). This test differs from the equivalency test because it examines whether the overlap between observed niches in two ranges is different from the overlap between the observed niche in one range and niches selected at random from the other range (Broennimann et al., 2012). We determined the invasive and native centroids of both niches and calculated the distance between each sampled individual and each centroid, and then values were averaged to obtain population‐level distance. Finally, environmental distance among populations was calculated using a niche plot.

### 2.5 Geographical and environmental drivers of genetic diversity

The relationship between genetic diversity (Ar) and Euclidian distance, environmental suitability, and distance to both invasive and native niche centroids was assessed using a bivariate linear regression model, whose *p*‐value was corrected using Holm–Bonferroni method correction. Moreover, we explored the relationship between genetic variation and both geographical and environmental distances using a Markov chain Monte Carlo (MCMC) with one million simulations, a thinning of 1000 simulations, beta_G from 2 to maximum geographical distance times 10 and beta_E from 1 to maximum environmental distance times 10, available in the *Sunder* package version 0.0.4 (Botta et al., 2015; Bradburd et al., 2013; Guillot et al., 2014; Guillot & Rousset, 2013). This approach uses a likelihood value to examine whether geographical distance, environmental distance, or a combination of both contributes to genetic differentiation (empirical covariance genotypes). In parallel, we estimated the isolation by distance (IBD), that is, the correlation between geographical distance and genetic differentiation (*F*_ST_) between populations (Wright, 1943) using a Mantel test based on Spearman's rank correlation rho and 10,000 simulations using *vegan* R package (Oksanen et al., 2022). Statistical significance was set at *p*‐value < .05.

## 3 RESULTS

### 3.1 Genetic structure and diversity

The amplification error rate of the microsatellite dataset was estimated to be 0.0015, based on the incorrect amplification of a single allele of the R4_100 microsatellite in a single sample (see Table S2 from Figshare in Data Accessibility and Benefit‐Sharing section). There were no signs of allelic dropout, however null alleles were detected for some loci in three populations (see Table S2 from Figshare). Quaresma et al. (2022) discuss that the microsatellite dataset of *V. velutina* generated in Europe may exhibit null alleles as an artefact resulting from combining samples collected across wide geographical areas and in different years, grouped by regions, causing a Wahlund effect. Following Quaresma et al. (2022), we did not discard any locus either and performed the ensuing analysis using the 15 loci.

**FIGURE 1:**
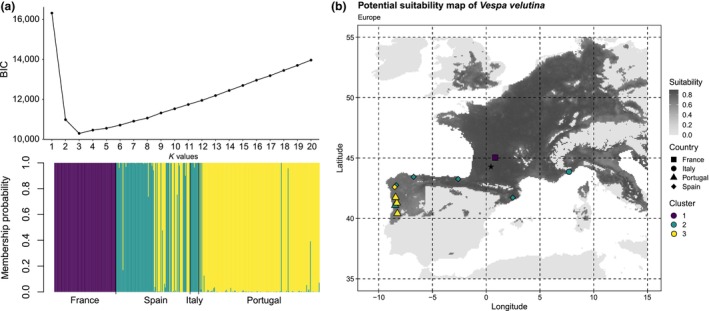
Genetic structure and environmental suitability of *Vespa velutina* in Europe. (a) Population admixture ancestry components are shown for the 357 individuals, where different countries are delimited by black borders. The lowest Bayesian information criterion (BIC) was obtained at *K* = 3. (b) Potential suitability is represented in a greyscale from dark grey (high values) to light grey (low values). The location of each population centroid included in the study and their genetic cluster are represented on the map. The black star represents the first field record of this species in Europe.

Three different genetic clusters were detected in Europe by structure analysis using the EM algorithm implemented in the *adegenet* package (Figure 1a). There was a clear and consistent genetic cluster in the French population (cluster 1, mean *Q*‐value = 100%), which was differentiated from the rest of the invasive populations in Europe. Six populations were assigned to cluster 2 (mean *Q*‐values: Catalonia = 93.75%, Basque Country = 100%, Asturias = 100%, Galicia = 44.23%, Italy = 100%, Portugal = 3.07%), and four populations to cluster 3 (mean *Q*‐values: Galicia = 55.77%, Portugal = 96.93%, which corresponds to Portugal N, C and S). Cluster 2 was the most widespread in southern Europe, ranging from Italy to Portugal. In contrast, cluster 3 was detected only in the westernmost part of the Iberian Peninsula. We calculated the genetic parameters for 11 different populations (Table 1). The highest diversity was detected in French population (Ar = 2.78), and the lowest in Portugal C population (Ar = 1.76) (Table 1). On the contrary, the lowest relatedness was detected in French population (Rnees = −0.05), and the highest in Portugal C population (Rness = 0.58) (Table 1).

### 3.2 Geographical peripherality and environmental suitability

After fitting and evaluating each single model 20 times using nine different methods, we obtained a final dataset of 180 models. A high weighted mean value using ROC scores (0.948) was observed for the final ensemble model, indicating good performance during model construction. The corresponding potential distribution identifies the centre of Europe, part of the Mediterranean coast, and the north and west of the Iberian Peninsula as high environmental suitability areas for *V. velutina* (Figure 1b). The environmental suitability values ranged between 0.610 (Portugal S cluster 3) and 0.869 (France cluster 1) for the populations included in this study (Table 1).

**FIGURE 2:**
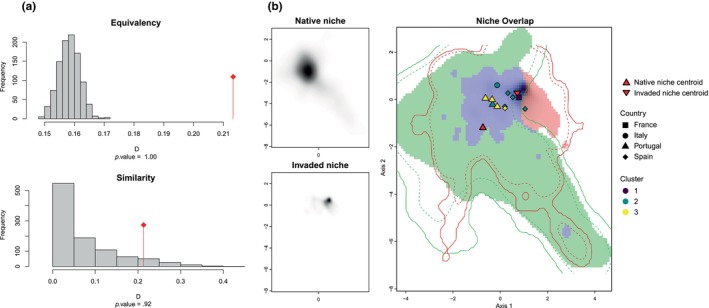
(a) Niche divergence test (equivalency and similarity tests) between invaded niche (red shading) and native niche (green shading) with observed niche overlap between the two ranges (bars with a diamond) and simulated niche overlaps (grey bars). Tests of niche equivalency and similarity were run using 1000 replications. (b) Actual density of occurrences in native and invaded niche, and overlap between niches are represented in blue, while solid and dashed lines show 100% and 75% of the available (background) environment respectively.

According to the niche divergence test carried out between both native and invaded distributional ranges, the invaded niche was not less equivalent to the native niche than random (*p*‐value = 1.00, Figure 2), likewise the similarity test showed that the invaded niche was not less similar to the native niche than expected by chance (*p*‐value = .92). Moreover, the niche centroid of *V. velutina* shifted from its native range to the invaded range in the same direction as the background (Figure 2). There was a niche overlap between both ranges of 21.33%, a niche expansion of 2.06%, a niche stability of 97.94%, and a niche unfilling of 48.49%. The French populations was the farthest away from the native centroid, and the rest of the populations were expanding towards the native niche centroid (Figure 2b). In this regard, the environmental distance to the native niche centroid ranged between 1.060 (Portugal cluster 2) and 1.987 (France cluster 1). On the contrary, French population was the closest from the invasive niche centroid, and Portuguese S population was the farthest away (Figure 2b). The distance to the invaded niche centroid ranged between 0.194 (France cluster 1) and 1.791 (Portugal S cluster 3) (Table 1).

**FIGURE 3:**
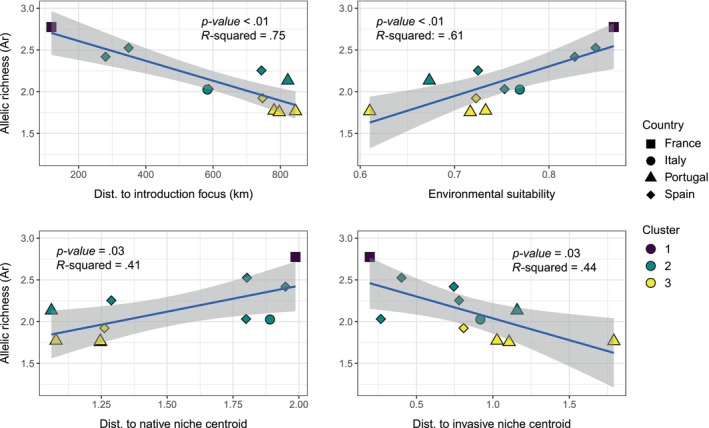
Bivariate linear regression models between genetic diversity and geographical and environmental drivers. The genetic parameters included allelic richness (Ar), while geographical and environmental drivers included distance to the introduction focus (km), environmental suitability and distance to both native and invaded niche centroids. *p*‐value was corrected using Holm–Bonferroni method correction.

**FIGURE 4:**
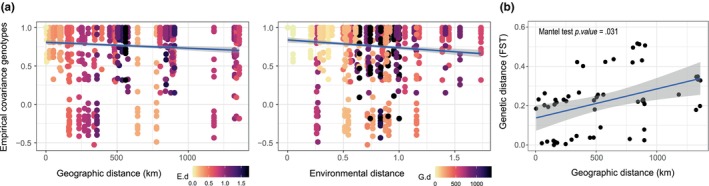
(a) Empirical covariance genotypes for all population pairs (*n* = 11) and loci (*n* = 75) based on the geographical distance (km) and environmental distance. Each graph is coloured by environmental distance (E.d) and geographical distance (G.d) respectively. (b) Isolation by distance (pairwise *F*_ST_ ~ Euclidean distance) plotted to compare populations.

We found a significant association between all pair comparisons: allelic richness (Ar) and distance to the introduction focus (km) (*p*‐value < .01, *R* ^2^ = .75), environmental suitability (*p*‐value < .01, *R* ^2^ = .61), distance to the native niche centroid (*p‐value* = .03, *R* ^2^ = .41), and distance to the invaded niche centroid (*p*‐value = .03, *R* ^2^ = .44) (Figure 3). Similarly, according to MCMC analysis, both geographical and environmental distances influenced population genetic differentiation (likelihood = −1486.72), rather than geographical distance (likelihood = −1490.63), or environmental distance separately (likelihood = −1529.16) (Figure 4a). This results agree with the isolation by distance analysis which was statistically significant (Mantel statistic *r*: .08, *p*‐value = .031) (Figure 4b).

## 4 DISCUSSION

Expansion range limits of species can be shaped by a combination of ecological and evolutionary factors, including demography, genetic diversity, habitat heterogeneity and gene flow (Sexton et al., 2009). A single introduction of *V. velutina* has been documented for Europe, with very few or possibly only one single multi‐mated female as the source of all genetic diversity on the continent (Arca et al., 2015). Moreover, according to Otis et al. (2023), *V. velutina* is the only *Vespa* species to date to present moderate polyandry, defined as 1–10 effective mates (Arca et al., 2015; Herrera et al., 2023; Jones et al., 2020), whereas *V. affinis*, *V. analis*, *V. crabro*, *V. dybowskii*, *V. mandarina* and *V. simillima* exhibit facultative low polyandry, and *V. ducalis* and *V. soror* are monoandrous. Furthermore, Favreau et al. (2023) showed evidence of selection pressure on *V. velutina* genes associated with reproduction, facilitating the potential transition from native to invasive ranges.

The genetic patterns observed for *V. velutina* seem to be associated mainly with the natural dispersion of the species throughout the invaded territory, following the chronology of the new records in European countries (Laurino et al., 2020). Evidence for natural dispersion comes from the recovery of new genetic clusters along the expansion front, with an exclusive genetic cluster in the founder population (cluster 1 in the French population) and in marginal populations (cluster 3 in Portuguese populations), and an intermediate genetic cluster with a more widespread distribution (cluster 2 in Italy, Spain, and Portugal). This scenario is most likely the result of cumulative genetic drift effects linked to successive founder events across the expansion range (Böhme et al., 2007), where newcomers are the offspring of the individuals living near the expansion front (Klopfstein et al., 2006).

Further support for a natural dispersion scenario comes from the gradual loss of allelic richness (Ar) as the front moves away from the introduction focus in France (Haxaire et al., 2006; Quaresma et al., 2022). However, human‐mediated dispersal as well as habitat disturbance have also been alluded to as beneficial for range expansion in this and other species of the genus *Vespa* (Alaniz et al., 2021; Monceau et al., 2014; Robinet et al., 2017). This is the case of the geographically isolated islands of Great Britain and Mallorca (Budge et al., 2017; Jones et al., 2020; Leza et al., 2021), in which *V. velutina* invasions resulted from human‐mediated dispersion, most probably due to shipping traffic from the mainland (Robinet et al., 2019). Long‐distance jumps have also been reported for *V. velutina* invasions on the continent, such as in Portugal (Quaresma et al., 2022). This combination of short and long distance dispersal is a common feature in several invasive insects (Gilbert et al., 2004; Meats & Smallridge, 2007; Sharov & Liebhold, 1998). While, long‐distance jumps may lead to a lack of relationship between genetic and spatial distances (Acevedo‐Limón et al., 2020), we detected that both geographical and environmental distances were influencing population genetic differentiation.

The constrained sample size presents a notable limitation in our research examining the central–marginal hypothesis through microsatellite analysis within European populations of an invasive species. Moreover, the unequal availability of environmental occurrences between native and invasive ranges may add uncertainty to overlap niche measurements (Beukema et al., 2018). Nonetheless, this unbalanced data have been widely used in niche modelling of other *Vespa* species (Lioy et al., 2023; Villemant et al., 2011; Zhu et al., 2020). Although our study has yielded significant findings and revealed consistent trends, it is important to carefully consider the potential effects and limitations associated with the small sample sizes we utilised. The small sample size may introduce biases and limit the ability to generalise our findings to the entire population of the invasive species. This highlights the need for future research with larger sample sizes to confirm and strengthen our observations. Similar patterns of genetic diversity have been observed in other studies with similar sample sizes (Garner et al., 2004; Ledoux et al., 2021).

The reduction of genetic diversity across the expansion range from the introduction focus has been well described in invasive species (van Boheemen et al., 2017) and it is here further documented for *V. velutina*, as revealed by the negative association between allelic richness and distance from the introduction focus. Interestingly, we also detected a positive association between allelic richness and environmental suitability, highlighting the importance of a suitable environment that enables the successful establishment of a diverse population. Hence, the low environmental suitability at the expansion limits could prevent a diverse population from establishing itself and reduce the genetic diversity in edge populations. Similar patterns have been reported for other invertebrate species (Acevedo‐Limón et al., 2020; Ortego et al., 2015). Unsuitable environmental conditions and lower genetic diversity in marginal populations translate into higher potential for genetic differentiation (Aguirre‐Liguori et al., 2017; Leimu & Fischer, 2008). Furthermore, distances to environmental niche centroids have been mentioned to reflect fitness‐related attributes, such as population abundance and genetic diversity (Martínez‐Meyer et al., 2013). In this study, we detected a positive association to native niche centroid, and a negative association to invasive niche centroid, reflecting a spatial pattern of genetic variation because environmental variables may impact population dynamics (Ochoa‐Zavala et al., 2022). Hence, there is a close link between the set of habitable conditions and geographical genetic variations, similarly to Lira‐Noriega and Manthey (2014).

Genetic drift can be an important force in shaping the genetic structure of expanding populations (Excoffier & Ray, 2008). For example, Bélouard et al. (2019) found that genetic drift dominated in the evolution of allele frequencies in isolated populations of the invasive red swamp crayfish during its spread in a wetland area. Furthermore, it seems that founder effects rarely limit the fitness of invasive alien insects and may even benefit populations (Garnas et al., 2016), although the detrimental impact of founder events on the genetic variability of edge populations (Sherpa et al., 2020) or on sex determination (Hagan & Gloag, 2021). The last one has been documented particularly on *V. velutina* (Darrouzet et al., 2015).

The relevance of the suitability–genetic diversity relationships for the management of biological invasions can be modulated by climate change. Barbet‐Massin et al. (2013) predicted a potential increase in environmental suitability and therefore a range expansion of *V. velutina* by 2100, towards the southwest region of the Iberian Peninsula. Based on our results, this potential expansion could lead to low genetic diversity at the expansion edges. Our findings could suggest that dispersal rates can evolve in response to the range edges where genetic diversity is lower. For instance, Simmons and Thomas (2004) observed increased frequencies of dispersive and long‐winged individuals in recently colonised bush cricket populations compared to longer‐established populations in the range core. However, after a decade, the individuals' wing morphology became similar to those of core populations. This pattern has also been observed in other insect species, such as butterflies (Hughes et al., 2003) and sand crickets (Roff & Fairbairn, 2007).

The empirical covariance genotype values from local (<100 km) to continental level (>1000 km) reflect the effect of geographical and environmental distances on genetic differentiation among *V. velutina* invasive populations. These results agree with the annual cycle and high dispersal rates registered for this species, ranging between 18 and 78 km per year in continental populations (Bertolino et al., 2016; Robinet et al., 2017). Based on previous discussion, historical processes seem to be the main factors shaping this pattern as for other invasive species (Ackiss et al., 2018; Chung et al., 2018; Eckert et al., 2008).

In summary, we found that populations at the edge of the *V. velutina* distributional range have lower allelic richness than populations at the range core. This pattern could have been shaped by life history, population sizes, genetic drift and gene flow among populations. Nonetheless, Arca et al. (2015) highlighted that other biotic and abiotic factors could compensate for the low genetic diversity in invading marginal populations, such as the abundance of honey bees or reduced competition with other hornets (Villemant et al., 2011). Hence, we cannot underestimate the invasiveness of this species on the European continent.

Our results highlight the importance of combining genetic analysis with geographical and environmental drivers to further understand the genetic patterns of *V. velutina* to the newly invaded environments. In addition, international coordination, and the implementation of preventative measures by all invaded countries are necessary to more efficiently control the spread of invasive species at the European level.

## AUTHOR CONTRIBUTIONS

**Cayetano Herrera:** Conceptualization (lead); data curation (lead); formal analysis (lead); investigation (lead); methodology (lead); software (lead); writing – original draft (lead). **M. Alice Pinto:** Data curation (supporting); investigation (supporting); methodology (supporting); supervision (equal); writing – review and editing (equal). **Mar Leza:** Data curation (supporting); investigation (supporting); methodology (supporting); supervision (equal); writing – review and editing (equal). **Iris Alemany:** Formal analysis (supporting); methodology (supporting); software (supporting); writing – review and editing (supporting). **José A. Jurado‐Rivera:** Data curation (supporting); investigation (supporting); methodology (supporting); supervision (equal); writing – review and editing (equal).

## CONFLICT OF INTEREST STATEMENT

The authors declare that they have no potential conflict of interest in relation to the study in this paper.

## Supporting information


Figure S1.

## Data Availability

Tables S1 and S2 from this publication are available in an open repository (Figshare). Link to the dataset and R codes are available at https://doi.org/10.6084/m9.figshare.22567267. Benefits from this research accrue from the sharing of our data and results on public databases as described above.
