# Supplementary material for: Niche modelling and landscape genetics of the yellow‐legged hornet (Vespa velutina): An integrative approach for evaluating central–marginal population dynamics in Europe
Source: Ecol Evol. 2024 Jul 24;14(7):e70029. doi: 10.1002/ece3.70029 (PMC11267635; doi:10.1002/ece3.70029)
Supplement: Supplementary file 1 — Figure S1. [file ECE3-14-e70029-s001.docx]

**Supplementary Materials**

| 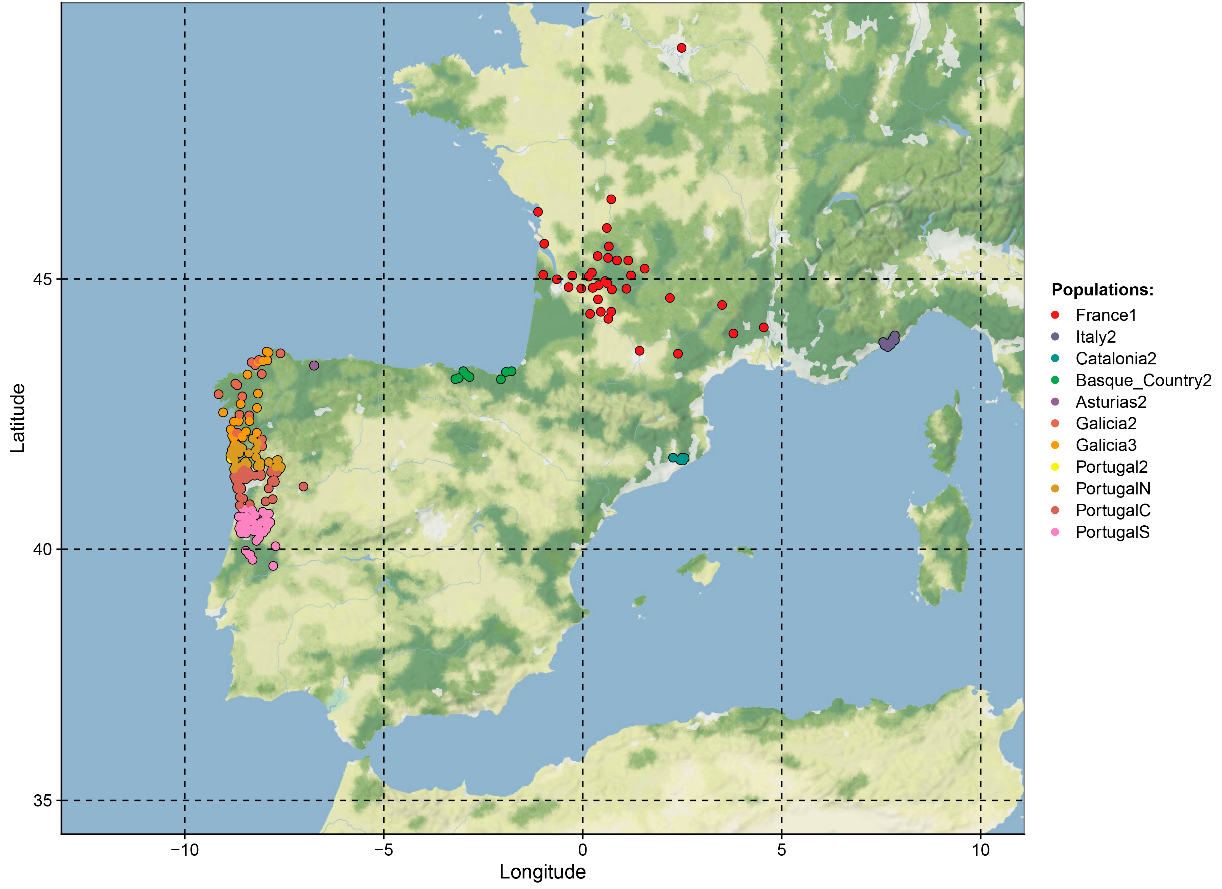 |
| --- |
| Figure S1.- Map of the individuals of the yellow-legged hornet *Vespa velutina* used in this study. Each colour represents individuals of each population with different its genetic cluster detected. Numbers following region codes refer to the genetic cluster detected (e.g., France1 stands for individuals from France where the genetic cluster 1 was detected). Moreover, genetic cluster 3 was detected in Portugal N (north), Portugal C (centre), and Portugal S (south). |
